# Simultaneous Determination of 18 Polycyclic Aromatic Hydrocarbons in Daily Foods (Hanoi Metropolitan Area) by Gas Chromatography–Tandem Mass Spectrometry

**DOI:** 10.3390/foods7120201

**Published:** 2018-12-08

**Authors:** Thanh-Thien Tran-Lam, Yen Hai Dao, Lien Kim Thi Nguyen, Hoi Kim Ma, Hai Nguyen Tran, Giang Truong Le

**Affiliations:** 1 Institute of Chemistry, Vietnam Academy of Science and Technology (VAST), 18 Hoang Quoc Viet, Cau Giay, Ha Noi 100000, Vietnam; thanhthien307@gmail.com (T.-T.T.-L.); dhy182@gmail.com (Y.H.D.); kimlien058@gmail.com (L.K.T.N.); 2 University of Science, Vietnam National University HCMC, Ho Chi Minh City 720040, Vietnam; makimhoi@gmail.com; 3 Institute of Research and Development, Duy Tan University, Da Nang 550000, Vietnam

**Keywords:** polycyclic aromatic hydrocarbons (PAHs), benzo(a)pyrene, QuEChERS, gas chromatography–tandem mass spectrometry (GC–MS/MS), daily foods, food processing

## Abstract

Polycyclic aromatic hydrocarbons (PAHs)—a large group of organic compounds—are extremely hazardous to human health. In this study, the 198 samples from six groups of daily food products in the Hanoi metropolitan area were collected and prepared by the QuEChERS sample treatment technique. The detection and identification of PAHs were obtained by gas chromatography–tandem mass spectrometry (GC–MS/MS) determination. The results demonstrated that the recovery of PAH compounds ranged approximately between 71% and 110% when the solvent evaporation condition was optimized using the nitrogen gas at a low temperature (1 °C). The in-house method was validated in terms of linearity, extractive condition, repeatability, recovery, limit of detection (LOD), and limit of quantification (LOQ). The ranges of average PAH levels were 9.3–9.6 µg/kg (for instant noodles), 0.22–2.48 µg/kg (for cakes) 0.91–4.83 µg/kg (dried vegetables), 5.14–23.32 µg/kg (teas), 4.82–24.35 µg/kg (coffees), and 1.43–25.2 µg/kg (grilled meats). The results indicated that the total concentrations of residual PAHs and benzo(a)pyrene in the instant noodles and grilled meat samples surpassed the maximum limits tolerated by the European Commission (35 µg/kg and 5 µg/kg, respectively) in many investigated samples.

## 1. Introduction

Food processing is frequently used to treat fresh foods (raw materials) in food products or transform treated raw materials into other forms of food. Cooking and processing food at a high temperature are acknowledged as important contributors to producing harmful toxics for human health [1]. Some toxins—heterocyclic amines (HCAs) and polycyclic aromatic hydrocarbons (PAHs)—can change the genome system and cause cancer [2]. These toxic agents, especially PAHs, are thus receiving attention worldwide [3].

In essence, PAHs, which are persistent organic pollutants, consist of numerous carbon atoms joined together to form multiple rings [4]. There are several hundred PAHs that usually occur as complex mixtures rather than as individual compounds [5]. Gaseous and particle-bound PAHs present in the atmosphere can be deposited and accumulated during vegetation growth [6,7]. When PAHs are adsorbed on the surface of dusts, they become highly thermally active and photosensitive and can be cracked at a high temperature. Furthermore, many PAHs have emerged as certain carcinogens in experimental animals [8] and have had a significant impact on the disease burden for various cancers in humans [3]. In mammalian cells, PAHs undergo metabolic activation to diol epoxides that covalently bind to cellular macromolecules (including DNA), causing the errors in DNA replication and mutation which initiate the carcinogenic process [9,10,11].

The most important anthropogenic sources of PAHs have been identified in the literature, such as coke ovens in the production of aluminium, iron, and steel [12,13], heating in power plants and residences [14], cooking, motor vehicle traffic [13], environmental tobacco smoke, and the incineration of waste material [15]. In the natural environment, PAHs might present as emerging contaminants in water bodies [16,17], vegetables, fruits, and cereals [18], and oils, fish, and meat [19,20]. Food-processing procedures—smoking, drying, and cooking—are generally considered to be the major source of PAH contamination in foods. The formation of PAHs in meat is often affected by several important factors, such as the methods used for food preparation, temperature, distance from the heat source, and the drainage of fat [21]. Tea and coffee can be contaminated by PAHs derived from environmental deposits [22] or heating steps [23,24,25]. Tea leaves have a large surface area where the accumulation of PAHs in vegetation can take place easily [22]. In addition, technical processes (i.e., withering followed by drying) can increase the concentrations of PAHs in tea leaves by more than 200 times [22,26]. Moreover, PAHs have been detected in instant noodles [27]. The production of PAHs in industries was reportedly favoured by an oxygen-deficient flame, temperatures in the range of 650–900 °C, and highly un-oxidized fuels [28]. The most toxicity-characterized carcinogenic PAHs are benzo(a)pyrene (B(a)P). B(a)P has been used as an indicator of the PAH content in food since 2002; the correlation coefficient between total PAHs and B(a)P concentration has been reported to be 0.87, and between the carcinogenic PAHs and B(a)P as 0.98 [21]. Furthermore, a maximum acceptable concentration of 5 µg/kg has been established by the European Commission for B(a)P in smoked meat and smoked meat products [29]. 

Some sample preparation methods—Soxhlet extraction, dispersed solid phase extraction (dSPE), and solvent centrifugal extraction coupled with gel permeation chromatography—have been extensively utilized to isolate PAHs from many kinds of matrices [30]. However, the application of such techniques needs to pass through several stages, which leads to some unexpected problems, such as possibly contaminated samples and false-positive results. In addition, these methods are not remarkably appropriate because of the utilization of numerous extraction solvents, taking more time, requiring more expensive equipment, and with a low efficiency of extraction. To overcome these challenges, the sample preparation method of QuEChERS (Quick, Easy, Cheap, Effective, Rugged, and Safe) was selected to determine PAH concentrations in food samples. This technique has been used for PAH detection in fat matrices, including fish [31] seafood [32], meat [32,33], and milk [34]. The previous studies indicated the ranges of recovery of 13 PAHs in smoked fish samples (97–120%), 12 PAHs in ham matrices (72–111%), 16 PAHs in grilled meat samples (71–104%), and 33 PAHs in salmon samples (70–120%). Furthermore, the outstanding advantages of the QuEChERS method are acknowledged to be as follows: (1) the performance obtains a high accuracy; (2) recovery ranges from 70% to 120%; and (3) the limit of detection (LOD) can be in the threshold of µg/kg or µg/L. Therefore, QuEChERS was selected to analyse PAHs in this study. This study aimed to simultaneously determine the PAH levels in daily foods available in the Hanoi area (the capital of Vietnam) by the QuEChERS method. On the basis of the obtained data, it is expected to evaluate the concentrations and the distributions of PAHs in different matrices.

## 2. Materials and Methods

### 2.1. Chemicals

The standard mixture of 18 PAHs in solvent acetonitrile: toluene (92:8) (EPA Method 8310 PAH Mixture, Restek, Bellefonte, PA, USA) includes acenaphthene, acenaphthylene, anthracene, benz(a)anthracene, benzo(a)pyrene, benzo(b)fluoranthene, benzo(g,h,i)perylene, benzo(k)fluoranthene, chrysene, dibenz(a,h)anthracene, fluoranthene, fluorene, indeno(1,2,3-cd)pyrene, 1-methylnaphthalene, 2-methylnaphthalene, naphthalene, phenanthrene, and pyrene. Isotope compounds were obtained from Dr. Ehrenstorfer GmbH (Augsburg, Germany), including the internal standards: benzo(a)anthracence-13C6, and benzo(g,h,i)pyrylene-13C12. All solvents (acetonitrile, *n*-hexane), which are of HPLC grade or pro analysis, were obtained from Fisher chemical (Pittsburgh, PA, USA). MgSO_4_ and NaCl were obtained from Merck. The sorbent Primary Secondary Amin (PSA) and octadecylsilane (C18) were purchased from Agilent (Santa Clara, CA, USA).

### 2.2. Equipment

GC–MS/MS analyses were performed on a Thermo Fisher Scientific (Waltham, MA, USA) system consisting of a Trace GC 1310 gas chromatograph, a TriPlus RSH Autosampler, and TSQ 8000 mass spectrometer (Thermo, Waltham, MA, USA). The TraceFinder software from Thermo Fisher Scientific was used for data processing. A DB5-MS (30 m × 0.25 mm, 0.25 µm) gas chromatography column was used to separate PAHs (Agilent, USA). Helium was used as a carrier gas at 1 mL/min. The following temperature program was used: isothermal at 70 °C for 1 min, then temperature was increased by 10 °C/min to 270 °C, then by 2 °C/min to 280 °C, then held for 3 min, then increased by 2 °C/min to 310 °C, and finally held for 1 min. The GC was interfaced by a heated transfer liner (310 °C) to the mass spectrometer in electron ionization mode with an electron energy of 70 eV. Nitrogen was used as the collision gas at the rate 1 mL/min. The criteria for the identification of PAHs were both the same retention times as the standard within ±2%, and correct relative abundance of two characteristic ions within ±15%. Identifying and quantifying ions, retention time, and collision energy are listed in Table 1. The chromatogram of 18 PAH compounds is illustrated in Figure S1.

### 2.3. Sample Collection

The samples were collected from March to June in 2018. The daily food samples (noodle, snack, dried vegetable, and coffee) were purchased from some supermarkets located in Hanoi, Vietnam (i.e., BigC Supercenter, Co.op Mart, and Metro Supermarket). Meanwhile, the tea samples were mainly purchased from some local markets in Hanoi, Vietnam (i.e., Cau Giay, Ha Dong, Hoang Mai, Long Bien, and Dong Da districts). The results of each group are shown in Table 2.

### 2.4. Sample Preparation

In essence, hexane and dichloromethane are non-polar solvents, so they are suitable for the characteristics of PAH. However, the two solvents are highly insoluble in water. Therefore, they are only utilized to extract PAHs on the matrix surface. In this study, the sample was prepared by the QuEChERS technique; as a result, acetonitrile and deionized water were used as solvents for extraction. Notably, the analytical samples are primarily analysed in the matrices that contain the high contents of starch, protein, and cellulose (see Section 2.3). Because acetonitrile is well-miscible in water, the combination of two solvents could deeply penetrate the structure of matrices. As a result, the PAHs can be effectively extracted. Furthermore, the composition of the selective samples includes oil and fat, so the matrices are also extracted when the used non-polar solvents, which results from the liner and source becoming contaminated during the analysis process [31,32,33,34].

According to the QuEChERS method [35,36], the following sample preparation was described as the follows. First, the samples were homogenized by grinding followed by the reduction using the quartering method. Meanwhile, the grilled meats had been firstly frozen before they were homogenized by grinding and reducing. Approximately 5 g of sample was transferred into a 50-mL Erlenmeyer flask. After that, 5 mL ultrapure-water and 10 mL acetonitrile were added into the flask, and the flask was shaken well for 1 min. Then, 4 g MgSO_4_ anhydrous and 1 g NaCl were added into the flask, and it was shaken for 3 min before centrifuging at 6000 rpm for 5 min. Next, 6 mL of supernatant was transferred to a 15-mL tube, and 0.9 g MgSO_4_, 0.3 g PSA, and 0.3 g C18 were added into the tube. The sample was vortexed for 3 min and centrifuged at 6000 rpm for 3 min. Then 3.00 mL supernatant was transferred to another tube and subsequently evaporated to a dry state by nitrogen at 1 °C using a nitrogen blowing system. The Julabo thermostat was used to stabilize the temperature of 1 °C. After that, 1.00 mL *n*-hexane was added to the tube, and the mixture was vortexed for 3 min. Finally, the resultant sample was purified by a 0.22-µm polytetrafluoroethylene (PTFE) filter before analysing.

Notably, during the sample treatment process, NaCl salt was used to increase the density of water supporting the extraction between the acetonitrile phase and water phase. However, NaCl will partially dissolve into the acetonitrile phase. With more solvent, NaCl will crystallize and non-dissolve again in *n*-hexane. In this case, the 0.22-µm PTFE filter was used to remove the crystalline NaCl and the suspended matter generated during the extraction. This avoids the crystallization of salt in the liner as well as the disable syringe.

### 2.5. Optimizing Solvent Evaporating Process

The standard PAH compounds were spiked into 3.00 mL of acetonitrile to achieve the contain of 10 µg/kg. Three different conditions were used for evaporating solvent. They include the use of nitrogen in the room temperature, nitrogen at 1 °C, and a vacuum rotating system. The additional samples after evaporating the solvent were dissolved and diluted to 1.00 mL.

### 2.6. Method Validation

Spiking was performed at 1 µg/kg with at least three series of duplicate blank sample to obtain the limit of detection as well as the repeatability. To achieve intermediate reproducibility, additional spiking at 5 and 10 of duplicate samples in three series was included. Recovery for the method was based on spiking at 10 µg/kg with six replicates. For each spiked sample, the individual PAH concentration was determined. The LOD was calculated as three times of the standard deviation on the calculated amount in each of the spiked samples.

## 3. Results and Discussion

### 3.1. Optimizing Solvent Evaporating Process

**Figure 1:**
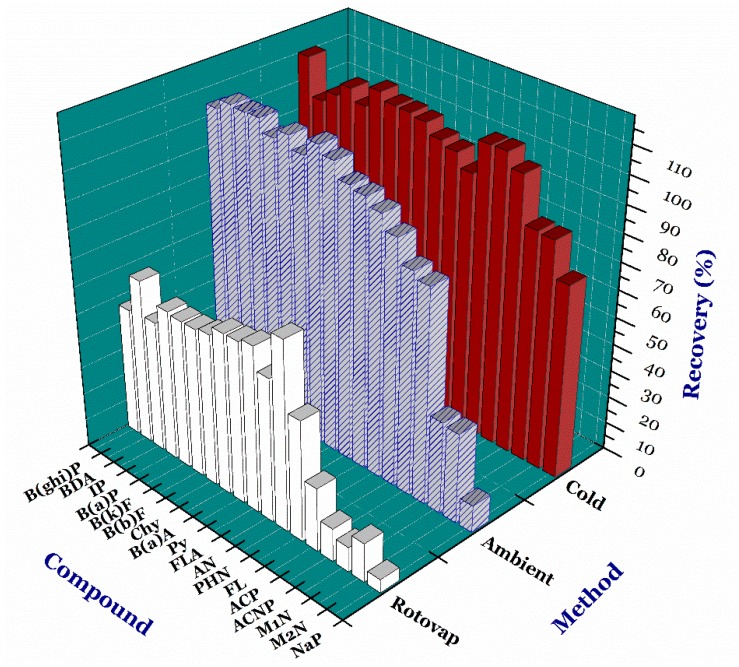
Comparison of recoveries between variable solvent evaporation methods. ***Note:*** naphthalene, NaP; 2-methylnaphthalene, M2N; 1-methylnaphthalene, M1N; acenaphthylene, ACNP; acenaphthene, ACP; fluorene, FL; phenalthrene, PHN; anthracene, AN; floranthene, FLA; pyrene, Py; benzo(a)athracene, B(a)A; chrysene, Chy; benzo(b)fluoranthene, B(b)F, benzo(k)fluoranthene, B(k)F; benzo(a)pyrene, B(a)P; indeno(1,2,3-cd)pyrene, IP; dibenz(a,h)anthracene, BDA; benzo(g,h,i)perylence, B(ghi)P; benzo(a)anthracence-^13^C_6_, B(a)A-13C6; and benzo(g,h,i)pyrylene-^13^C_12_,B(ghi)P-^13^C_12_.

The sample preparation process was based on the QuEChER extraction method requiring the utilization of acetonitrile solvent to enhance efficiency of the extraction of analytical substances from the sample matrix. However, acetonitrile is not favourable as an analytical solvent. This means that acetonitrile (the intermediate polar solvent) was not used as injection solvent in this study. Moreover, PAHs are volatile compounds, and thus the solvent evaporation easily leads to sample loss. For this reason, we compared the recovery efficiency of PAHs by three methods used to evaporate the solvent. They include the use of vacuum rotating system (named as the rotovap method), nitrogen gas at a low temperature (1 °C; the cold method), and nitrogen gas at a room temperature (25 °C; the ambient method) with a flow rate of nitrogen of 5 mL/min. The results are summarised in Table S1 and Figure 1.

For the rotovap method, the PAH compounds were volatilized easily and rapidly (the recoveries are approximately from 5% to 70%). In contrast, the cold method demonstrated the optimum results (the recoveries of 18 PAH compounds as expected in the range of around 71% to 110%). Meanwhile, the ambient method indicated the low recoveries of naphthalene (NaP; 10.04%), 1-methylnaphthalene (M2N; 33.17%), and 1-methylnaphthalene (M1N; 34.02%). In fact, at 25 °C, substances have a strong volatility (the vapor pressure from 9 Pa to 12 Pa). In addition, a high temperature (approximately 30 °C) and consecutive flow of nitrogen gas possibly contributed to increasing the volatility of these substances. Therefore, the volatilization of the solvent at the low temperature combined with the nitrogen assistance resulted from an excellent recovery (>84%; with the exception of NaP having the recovery =71%) and a minimal sample loss of the compounds. Hence, the cold method was selected to prepare selective samples in this study.

### 3.2. Method Validation

The limit of detection (LOD) and the limit of quantification (LOQ) of 18 PAHs ranged from 0.01 to 0.20 µg/L and from 0.03 to 0.60 µg/L, respectively. In order to meet recent EU regulatory requirements for the analysis of these 18 PAHs, the LOD should be lower than 0.3 µg/L. An acceptable intermediate reproducibility of 5–20% relative standard deviations (RSD_r_) was found to be within the criterion of RSD_r_ < 23%. The repeatability relative standard deviation (RSD_R_) was acceptable within the criterion of RSD_R_ < 23% (Table 3).

### 3.3. Recovery

The standard addition method was used to evaluate the recovery of PAHs. The standard of 18 PAHs and the isotopic standards of benzo(a)anthracence-13C6 and benzo(g,h,i)pyrylene-13C12 at concentration of 10 µg/mL were diluted by adding acetonitrile to 100 ng/mL, followed by adding water to provide the blank samples with a target concentration of 10 ng/mL. Accurate 5.00 mL of this standard solution was subjected into the sample treatment processing. The isotopic standards were used during the study to assess the recovery of the method and estimate the background effect as well as control the uncertainty of the GC–MS/MS analysis. 

**Figure 2:**
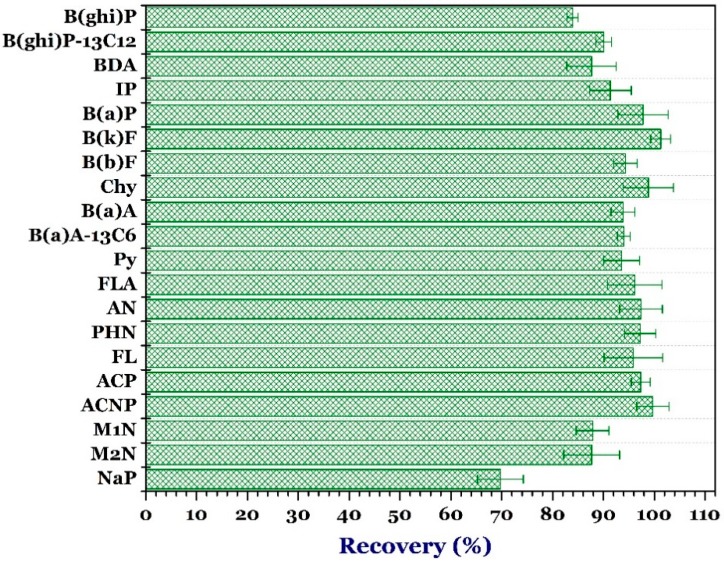
Recovery percentage of 20 compounds of polycyclic aromatic hydrocarbons. ***Note:*** naphthalene, NaP; 2-methylnaphthalene, M2N; 1-methylnaphthalene, M1N; acenaphthylene, ACNP; acenaphthene, ACP; fluorene, FL; phenalthrene, PHN; anthracene, AN; floranthene, FLA; pyrene, Py; benzo(a)athracene, B(a)A; chrysene, Chy; benzo(b)fluoranthene, B(b)F, benzo(k)fluoranthene, B(k)F; benzo(a)pyrene, B(a)P; indeno(1,2,3-cd)pyrene, IP; dibenz(a,h)anthracene, BDA; benzo(g,h,i)perylence, B(ghi)P; benzo(a)anthracence-^13^C_6_, B(a)A-13C6; and benzo(g,h,i)pyrylene-^13^C_12_,B(ghi)P-^13^C_12_.

Figure 2 shows that the recovery of 18 PAHs ranged approximately from 70% to 101%. Some PAHs (i.e., NaP, M2N, and M1N) had a low recovery because they are volatile compounds. In addition, the high molecular weight compounds (i.e., indeno(1,2,3-cd)pyrene (IP), dibenz(a,h)anthracene (BDA), and benzo(g,h,i)perylence (B(ghi)P)) indicated a low recovery efficiency due to the results of using C18 to eliminate and absorbing fat available in the samples. The high molecular weight can be also adsorbed by C18. Notably, the recovery of isotopic standards was more than 90% and did not indicate the loss of sample. For the identification purpose of multiple compounds simultaneously in one analytical process, the recovery of the substances exceeded 80% (exception for NaP recovered at 70%). As expected, the process was highly quantitative, enabling the identification and quantification of the concentration of PAHs in the food samples.

### 3.4. Levels of PAHs in the Target Samples

#### 3.4.1. Instant Noodle

**Figure 3:**
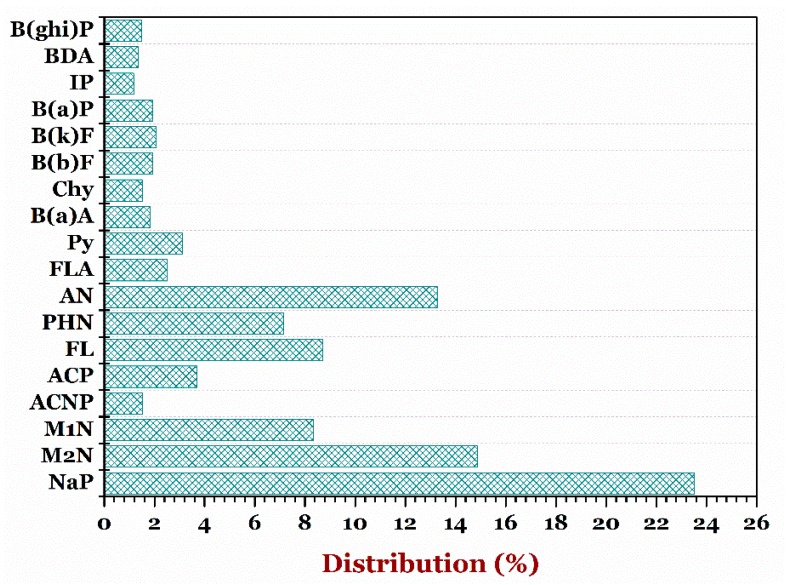
Distribution percentage of diffident kinds of polycyclic aromatic hydrocarbons in the instant noodle samples. ***Note:*** naphthalene, NaP; 2-methylnaphthalene, M2N; 1-methylnaphthalene, M1N; acenaphthylene, ACNP; acenaphthene, ACP; fluorene, FL; phenalthrene, PHN; anthracene, AN; floranthene, FLA; pyrene, Py; benzo(a)athracene, B(a)A; chrysene, Chy; benzo(b)fluoranthene, B(b)F, benzo(k)fluoranthene, B(k)F; benzo(a)pyrene, B(a)P; indeno(1,2,3-cd)pyrene, IP; Dibenz(a,h)anthracene, BDA; and benzo(g,h,i)perylence, B(ghi)P.

The 18 PAHs in the 65 samples of the instant noodles from the 13 different producers were determined by the validated GC–MS/MS analysis. Figure 3 displays the PAHs occurrence data provided for whole instant noodles obtained from the 13 different producers (*n* = 65). The result demonstrated that NaP (235.6 µg/kg) had the highest proportion of PAHs, followed by M2N (182.81 µg/kg) > AN (54.12 µg/kg) > M1N (35.01 µg/kg) > ACP (27.2 µg/kg). In contrast, low concentrations (from non-detection µg/kg to 11.90 µg/kg) were determined in some certain PAHs, such as B(a)P, IP, BDA, and Chy (Table 2). They are classified as probable human carcinogens. However, the result was inconsistent with the findings of Charles and co-workers [27].

Furthermore, B(a)P was detected in the 44/65 samples of collected instant noodles. Among the 44 samples, a higher concentration of B(a)P than the standard concentration from the European Commission (EC) rules (5 µg/kg) was identified in the 7/44 samples. Furthermore, as showed in Table 2, the concentrations of PAHs (ND—182.8 µg/kg) and B(a)P (ND—11.9 µg/kg) in the fried noodles were overwhelmingly higher than those in the non-fried noodles (ND—57.2 µg/kg and ND—6.6 µg/kg), respectively. This is because the difference of the raw materials’ properties and processing technologies between the fried noodles and the non-fried noodles.

#### 3.4.2. Cake and Dried Vegetable

**Figure 4:**
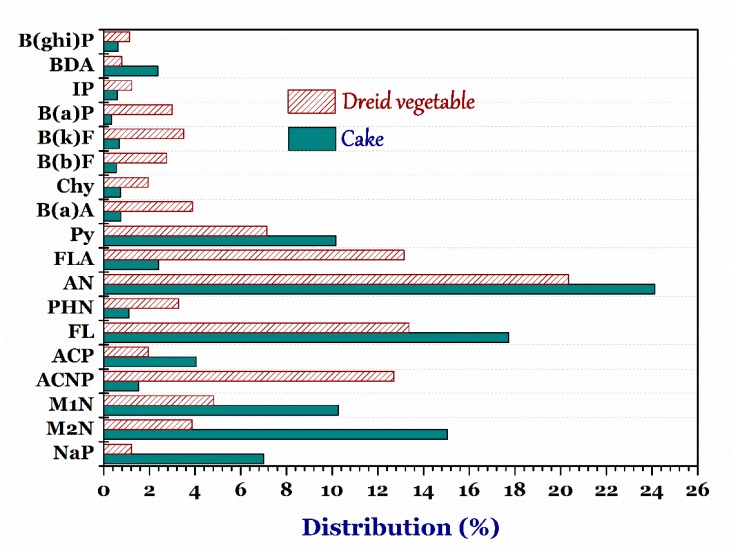
Distribution percentage of diffident kinds of polycyclic aromatic hydrocarbons. ***Note:*** naphthalene, NaP; 2-methylnaphthalene, M2N; 1-methylnaphthalene, M1N; acenaphthylene, ACNP; acenaphthene, ACP; fluorene, FL; phenalthrene, PHN; anthracene, AN; floranthene, FLA; pyrene, Py; benzo(a)athracene, B(a)A; chrysene, Chy; benzo(b)fluoranthene, B(b)F, benzo(k)fluoranthene, B(k)F; benzo(a)pyrene, B(a)P; indeno(1,2,3-cd)pyrene, IP; dibenz(a,h)anthracene, BDA; and benzo(g,h,i)perylence, B(ghi)P.

Figure 4 indicates that the PAHs content was partly dependent on the processing methods and characteristics of raw materials. PAHs in the cake primarily exist in light molecular weight and low toxicity, while the opposite was true for PAHs in the dried vegetables (heavier molecular weight and higher toxicity) [37]. In the cake samples, the ranges of average concentrations (*n* = 26) of PAHs and B(a)P were 0.22–2.48 µg/kg and 0.34–0.93 µg/kg, respectively (Table 2). The total high PAH contents were found in the starch-containing samples (the cakes) and oil-dried process at a high temperature (>200 °C) within an oxygen-deficient environment. The EC Regulation (No 1881/2006) has set the maximum allowed level (MLS) for B(a)P in cereal-based food as 1.00 µg/kg [38]. The maximum average distribution of B(a)P content (0.93 µg/kg) was lower than EC standard (equalizing to EC LOD value). Specifically, the average distribution of B(a)P content accumulated in the oil-dried samples (0.71–0.93 µg/kg) was significantly higher than that in the electric stove-dried samples (0.34 µg/kg). Notably, the concentrations of PAHs (ND–26.92 µg/kg) and B(a)P (ND–1.33 µg/kg) determined in the collected cake samples were remarkably lower than the corresponding values in the literature. For example, Iwegbue et al. [39] reported the total contents of 16 PAHs in many kinds of cakes (18.4–880.3 µg/kg), as well aschrysene content (0.1–66.4 µg/kg), and B(a)P content (0.1–50 µg/kg). The dissimilar results resulted from the different baking methods, fuel types, raw materials, and baking temperatures. Furthermore, individual PAH percentages were indirectly proportional to the ring numbers of PAH compounds. For example, the three-ring and four-ring PAH compounds primarily include phenanthrene, fluoranthene, and pyrene. The concentrations of three compounds increased when the baking temperature was higher than 220 °C [23]. Moreover, a high baking temperature (260 °C) caused an increase in the appearance of pyrene, chrysene and benzo(a)anthracene [39]. In fact, when the baking temperature increased, the transformation of low molecular weight PAHs into high molecular weight ones also increased [23].

In the dried vegetable samples, the PAHs and B(a)P contents did not indicate remarkable differences among the food types of samples because of interference in the living environment of the plants. Moreover, the average B(a)P contents in the dried jackfruit (0.52 µg/kg) and dried potato (1.20 µg/kg) were higher than the corresponding values in the 200 food items reported by Kazerouni et al. [21]. They reported that the highest B(a)P levels of collards and kale were 0.48 µg/kg and 0.47 µg/kg, respectively. Notably, the highest B(a)P contents in the dried sweet potato and dried vegetable were detected to be 2.39 µg/kg and 1.22 µg/kg, respectively. Some researchers also reported that vegetables (exception for potato) exposed to the polluted air contained high PAH levels [40]. Additionally, as reported by Greenberg et al. [41], it was recognizable that leaf vegetables possessed a higher PAH content than their tubers and fruits. Therefore, the dried vegetables contained low PAHs concentrations because of the contaminations from the environment along with the processing method.

#### 3.4.3. Tea and Coffee

**Figure 5:**
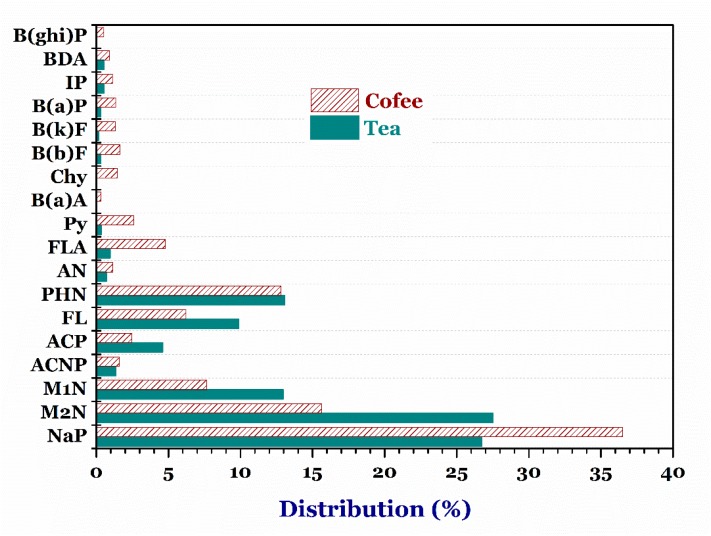
Distribution percentage of individual PAH in the tea and coffee samples. ***Note:*** Naphthalene, NaP; 2-methylnaphthalene, M2N; 1-methylnaphthalene, M1N; acenaphthylene, ACNP; acenaphthene, ACP; fluorene, FL; phenalthrene, PHN; anthracene, AN; floranthene, FLA; pyrene, Py; benzo(a)athracene, B(a)A; chrysene, Chy; benzo(b)fluoranthene, B(b)F, benzo(k)fluoranthene, B(k)F; benzo(a)pyrene, B(a)P; indeno(1,2,3-cd)pyrene, IP; dibenz(a,h)anthracene, BDA; and benzo(g,h,i)perylence, B(ghi)P.

A huge number of the PAH compounds with small molecular weights were chiefly found in the tea and coffee samples (Figure 5). This is because the production process partly contributed to simulating residual PAHs in the samples. For example, Table 2 shows that the average PAHs and B(a)P contents in the tea samples ranged from 5.14 to 23.32 µg/kg and from 0.91 to 9.42 µg/kg, respectively. The highest total PAHs contents and B(a)P contents were found in the black teas, while the lowest corresponding contents were found in the green teas. Notably, the B(a)P content in one black tea sample (19.82 µg/kg) exceeded the EC standard. The high PAH concentration detected in the black tea resulted from the processing section. Especially, tea leaves were directly exposed to smoke generated from wood-burning facilities during the distinctive drying section.

Notably, the European Commission fixed the limits for B(a)P and PAH4 in different foodstuffs (with the exception of tea) in amendment 835/2011/EC. Therefore, it is necessary to compare the result of the current study with those of previous studies. PAHs are abundant in the atmosphere and ubiquitous in several tea-growing regions, such as India [42], Sri Lanka [43], China [44], and Tibet [45]. According to Schlemitz and Pfannhauser [46], the PAH concentrations in different types of tea brand names commonly ranged between 4.9 and 103.6 µg/kg; the B(a)P contents were reported as 0.44 µg/kg for Assam tea, 5.52 µg/kg for Earl Grey tea, and 1.34 µg/kg for Ceylon tea. Furthermore, other scholars reported high B(a)P contents found in the tea samples, such as in black tea samples (4.1–32 µg/kg) [47], Assam black tea (152 µg/kg), Czech Republic green tea (17.9 µg/kg), Chinese black tea (246 µg/kg), and Chinese green tea (31 µg/kg) [48]. Another study reported on the B(a)P contents in Japanese green tea (3.8 µg/kg), Chinese green tea (5.0 µg/kg), Oolong tea (5.8 µg/kg), and Kayuaro black tea (73.2 µg/kg) [49]. Clearly, some of these reported concentrations exceeded the limits of PAH content in foodstuffs established by Regulation 835/2001/EC, namely 6 µg/kg for B(a)P. 

Furthermore, the ranges of average contents in the coffee samples were between 5.24 and 24.35 µg/kg for PAHs and between 0.31 and 9.16 µg/kg for B(a)P (Table 2). The B(a)P data in the coffee samples were overwhelmingly higher than the corresponding values of other scholars, such as 0.1–0.4 µg/kg [50] and 0.18–0.52 µg/kg [51]. Notably, the highest PAHs (ND–119.1 µg/kg) and B(a)P (4.52–21.35 µg/kg) concentrations were found in the instant coffee samples. In the roasted coffee, the roasting process had a strong effect on PAHs contents because this process produced more smoke through high-temperature heat release. Moreover, the low average B(a)P contents in the Arabica and Robusta coffees were 1.21 µg/kg and 0.31 µg/kg, respectively. In contrast, the B(a)P contents in the Cherry coffee reached up to 4.52 µg/kg. Similarly, the PAHs concentrations in the Cherry coffee were much higher than the Arabica and Robusta coffees (Table 2). This is because many supplemental materials (i.e., butter and odorants) were further added during the processing procedure, which leads to increasing adsorption of PAHs. 

#### 3.4.4. Grilled Meat

**Figure 6:**
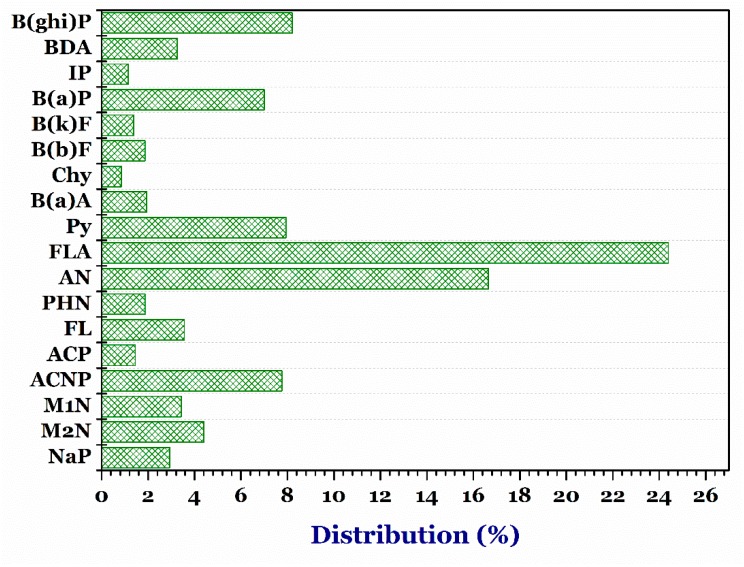
Distribution percentage of individual PAHs in the grilled meat samples.

All types of grilled meats after purchasing were standardized and stored at −18 °C. The grilled meat samples are classified into the following categories (four methods): the grilling of meat using stone coal (coal-grilling), the grilling of silver aluminium foil-wrapped meat using stone coal (wrapped coal-grilling), the grilling of meat using hardwood charcoal (charcoal-grilling), and the grilling of meat using an electric stove (stove-grilling). The analysis results are summarised in Table 2 and Figure 6. The results demonstrated the average PAHs and B(a)P concentrations in the range from 1.43 to 25.2 µg/kg and from 1.08 to 49.15 µg/kg, respectively. The types of large molecular weight PAHs were mostly presented, especially the B(a)P content accounting for approximately 7%. The processing section had a straight impact on the formation of PAHs in the food samples. 

The occurrence of B(a)P content in the grilled meats was strongly dependent on the grilling methods. In general, the highest B(a)P content was found in directly grilling on stone coal and charcoal, while the lowest was observed in grilling by the electric stove. The PAHs contents decreased in the following order: coal-grilling > charcoal-grilling > wrapped-coal-grilling > stove-grilling. For the wrapped-coal-grilling, the amount of grease released during the grilling process was restricted to directly contact with the smoke naturally generated from coal. In contrast, using electric stove to cook meat (stove-grilling) can prevent the meat from the exposition of strong PAHs emission sources (i.e., stone coal and charcoal). As a result, the stove-grilling method produced less PAHs levels for food.

Notably, the grilled meat samples prepared the coal-grilling and the charcoal-grilling methods exhibited approximately 10-times and 5-times higher B(a)P concentrations compared to the EC standard, respectively. In addition, the meat grilled with electric stove (the stove-grilling) indicated the lowest total PAHs and B(a)P contents. According to Chen and Lin [52], the PAH concentrations contained in the grilled meat samples treated with stone coal were much higher than those with heat produced by gas. Madusa et al. [53] proved that the grilled meat using electric stove generated less PAHs levels than that cooking flame because the fat contents were restrained to contact with the flame, and the combustion did not happen [1,54,55,56,57].

#### 3.4.5. PAHs in Daily Foods

**Figure 7:**
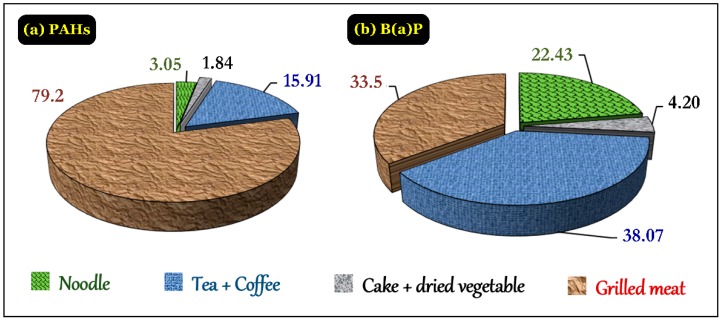
Distribution percentages of (**a**) PAHs and (**b**) benzo(a)pyrene (B(a)P) in the food samples.

The processing techniques and procedures strongly affected the PAHs contents in the foodstuffs. The PAHs content was primarily detected in the target samples that were directly processed with heat, such as the grilled meat, tea, and coffee samples (Figure 7a). The low-fat samples exhibited the lowest PAHs and B(a)P contents because they were primarily treated by the virtue of drying. Food roasted or fried at high temperatures in oxygen-limited conditions revealed a PAHs infection situation. Food with high fat or directly exposed to PAH emission sources (like tea and coffee), or by both factors (such as grilled meat) had high PAHs and B(a)P concentrations, exceeding the EC threshold (Figure 7b). Moreover, the environmental impact of PAHs such as materials for processing food also affected the concentration of PAHs and B(a)P. Thus, it is recognizable that the processing methods are likely to produce toxins harming human health. 

## 4. Conclusions

The levels of 18 PAHs in daily foods in the Hanoi area using the analytical methods in this study met the criteria of limit of detection (LOD) and recovery, as described by European Commission Regulation No. 836/2011 (such as, LOD < 0.3 µg/kg and recovery between 50% and 120%).

The analytical results confirmed that the PAHs contamination in the selected foods was engendered by the fundamental characteristics of foods and the food processing methods. The processing methods that caused the PAHs contamination of food included frying in oil and roasting, and particularly drying and grilling. We demonstrated that grilled meat sample processing with coal (coal-grilling) resulted in the highest average PAHs concentrations, such as at 49.15 µg B(a)P/kg and 25.2 µg PAH/kg. In contrast, the selected vegetable and cake samples processed by the drying method indicated low PAH contents, with the B(a)P content being lower than the EC threshold. The collected tea and coffee samples were directly influenced by drying on a flame during the processing procedure. The contents of PAHs in the tea samples exhibited the following decreasing order: black tea > Oolong tea > green tea. For the grilled meat samples, the grilled meat that was directly cooked on stone coal or had added spices of unknown origin had increased the PAHs concentrations detected. It can be concluded that the PAHs and B(a)P contents in the foods are strongly dependent on raw materials, safe processing methods, and clear origins and sources. 

## Figures and Tables

**Table 1:** Identifying, quantifying ions, retention time, and collision energy of 20 polycyclic aromatic hydrocarbons (PAHs).

Abbreviations	Compound	*t*_R_ (min)	Prec. (m/z)	Frag. (m/z)	CE (eV)	Remark
NaP	Naphthalene	7.1	128.2	127.2 (102.1)	15 (20)	Q (C)
M2N	2-Methylnaphthalene	8.63	141.1	89.1 (115.1)	16 (14)	Q (C)
M1N	1-Methylnapththalene	8.87	141.1	89.1 (115.1)	32 (15)	Q (C)
ACNP	Acenaphthylene	10.69	154.1	153.0 (152.0)	15 (20)	Q (C)
ACP	Acenaphthene	11.14	152.1	151.0 (150.0)	15 (20)	Q (C)
FL	Fluorene	12.32	166.1	154.0 (164.0)	15 (20)	Q (C)
PHN	Phenalthrene	14.55	178.2	176.0 (172.0)	15 (20)	Q (C)
AN	Anthracene	14.65	178.1	176.1 (152.1)	15 (20)	Q (C)
FLA	Floranthene	17.38	202.1	200.1 (152.1)	15 (20)	Q (C)
Py	Pyrene	17.88	202.1	200.2 (152.1)	15 (20)	Q (C)
B(a)A	Benzo(a)athracene	20.76	228.1	226.1 (202.2)	30 (35)	Q (C)
Chy	Chrysene	20.84	228.1	226.2 (202.1)	30 (35)	Q (C)
B(b)F	Benzo(b)fluoranthene	23.6	252.2	250.0 (226.0)	25 (30)	Q (C)
B(k)F	Benzo(k)fluoranthene	23.6	252.2	250.1 (226.1)	25 (30)	Q (C)
B(a)P	Benzo(a)pyrene	24.41	252.0	250.1 (226.1)	25 (30)	Q (C)
IP	Indeno(1,2,3-cd)pyrene	28.77	276.0	274.1 (250.0)	30 (40)	Q (C)
BDA	Dibenz(a,h)anthracene	28.99	278.2	276.1 (252.1)	30 (40)	Q (C)
B(g,h,i)P	Benzo(g,h,i)perylence	29.91	276.1	274.1 (250.1)	30 (40)	Q (C)
B(a)A-13C6	Benzo(a)anthracence-^13^C_6_	20.76	234.1	232.1 (208.1)	30 (35)	Q (C)
B(ghi)P-13C12	Benzo(g,h,i)pyrylene-^13^C_12_	29.91	288.2	286.2 (261.2)	30 (40)	Q (C)

The values in parentheses represent confirmation (C). Abbreviations: retention time (*t*_R_), precursor ion (Prec.), fragment ion (Frag.), quantitation (Q), confirmation (C), and collision energy (CE).

**Table 2:** The concentrations (µg/kg) of benzo(a)pyrene (B(a)P), chrysene, non-carcinogenic PAHs, and total 18 PAHs in 198 samples.

Food Types	*n*	B(a)P	Chrysene	Non-Carcinogenic PAHs	18 PAHs
**1. Instant noodles**					
Fried noodles	45	ND–11.9 (3.6)	ND–11.0 (2.9)	ND–182.8 (14.8)	ND–182.8 (9.6)
Non-fried noodles	20	ND–6.6 (2.7)	ND–5.9 (2.0)	ND–57.2 (15.1)	ND–57.2 (9.3)
**2. Cakes**					
Snack (fried)	7	ND–2.9 (0.93)	0.13–0.92 (0.64)	ND–11.99 (2.78)	ND–11.99 (1.74)
Snack (non-fried)	4	ND–0.65 (0.25)	0.04–0.30 (0.16)	ND–1.99 (0.21)	ND–2.32 (0.22)
Cookie (fried)	6	0.10–1.33 (0.71)	1.09–3.11 (2.12)	ND–26.92 (3.33)	ND–26.92 (2.21)
Cookie (non-fried)	5	ND–0.56 (0.34)	ND–1.49 (0.64)	ND–23.94 (2.83)	ND–23.94 (1.72)
Fritter	4	ND–0.83 (0.34)	ND–0.19 (0.12)	ND–13.30 (3.98)	ND–13.30 (2.48)
**3. Dried vegetables**					
Dried potato	5	ND–2.39 (1.20)	0.11–0.56 (0.22)	ND–8.22 (2.12)	ND–8.22 (1.93)
Dried carrot	4	ND–2.11 (0.82)	0.15–3.21 (1.07)	ND–8.61 (1.92)	ND–8.61 (1.72)
Dried sweet potato	5	ND–1.92 (0.81)	ND–1.81 (0.81)	ND–13.30 (3.12)	ND–13.3 (2.93)
Dried vegetable	5	ND–1.22 (0.75)	ND–1.52 (0.75)	ND–10.50 (5.06)	ND–10.5 (4.83)
Dried jackfruit	6	ND–1.04 (0.52)	ND–1.17 (0.56)	ND–5.09 (1.03)	ND–5.09 (0.91)
**4. Teas**					
Green tea (TN)	5	0.21–2.2 (1.03)	1.09–2.71 (1.43)	ND–71.62 (9.24)	ND–71.6 (8.12)
Green tea (LD)	5	0.14–1.82 (0.91)	0.72–3.72 (1.12)	ND–81.2 (8.79)	ND–81.2 (5.14)
Oolong tea (TN)	7	2.12–4.23 (3.21)	3.21–8.32 (5.32)	2.08–144.3 (38.12)	2.08–144.3 (23.08)
Oolong tea (LD)	5	1.01–2.95 (1.32)	ND–6.63 (2.42)	ND–90.2 (21.13)	ND–90.2 (12.45)
Black tea	7	4.89–19.82 (9.42)	7.64–17.2 (9.21)	1.12–199 (37.91)	0.912–318 (23.32)
**5. Coffees**					
Arabica	5	ND–1.51 (1.21)	ND–2.13 (1.12)	ND–37.23 (6.18)	ND–37.23 (5.24)
Robusta	7	ND–1.34 (0.31)	ND	ND–30.32 (5.21)	ND–30.32 (4.82)
Cherry	3	2.31–6.72 (4.52)	1.43–3.92 (2.59)	ND–72.52 (16.32)	ND–72.52 (15.11)
Instant coffee	9	4.52–21.35 (9.16)	2.89–10.12 (7.26)	ND–119.10 (26.32)	ND–119.1 (24.35)
**6. Grilled meats**					
Coal-grilled	7	23.12–65.3 (49.15)	2.13–5.62 (3.31)	4.84–160.03 (30.41)	2.13–160.03 (25.2)
Wrapped coal-grilled	3	5.82–15.62 (10.41)	1.23–3.01 (2.41)	1.72–59.3 (13.12)	1.23–59.3 (12.42)
Charcoal 1, grilled	7	19.24–30.25 (26.1)	3.65–12.42 (6.02)	4.52–112.6 (24.85)	3.65–112.6 (23.83)
Charcoal 2, grilled	7	13.19–28.45 (23.4)	4.45–10.72 (8.57)	0.31–116.21 (18.74)	0.31–116.2 (18.45)
Stove-grilled	5	ND–4.23 (1.08)	ND–0.23 (0.14)	ND–3.61 (1.52)	ND–3.61 (1.43)

The values in parentheses represent the average value of the compound concentration range. Abbreviations: non–detected (ND), number of samples (*n*), Thai Nguyen province (TN), Lam Dong province (LD). Non-carcinogenic PAHs (polycyclic aromatic hydrocarbons) include naphthalene, acenaphthylene, acenaphthene, fluorene, phenanthrene, anthracene, fluoranthene, and pyrene.

**Table 3:** Limit of detection (LOD; µg/kg), limit of quantification (LOQ; µg/kg), repeatability relative standard deviation (RSD_r_; *n* = 8), and reproducibility relative standard deviation (RSD_R_; *n* = 12).

Abbr.	Compounds	LOD (µg/kg)	LOQ (µg/kg)	RSD_r_ (%)	RSD_R_ (%)
NaP	Naphthalene	0.10	0.30	20	17, 10, 20
M2N	2-Methylnaphthalene	0.20	0.60	18	16, 9, 11
M1N	1-Methylnapththalene	0.05	0.15	16	19, 13, 18
ACNP	Acenaphthylene	0.05	0.15	12	18, 6, 14
ACP	Acenaphthene	0.05	0.15	10	16, 5, 13
FL	Fluorene	0.01	0.03	14	8, 9, 15
PHN	Phenalthrene	0.05	0.15	8	13, 9, 18
AN	Anthracene	0.05	0.15	13	16, 7, 17
FLA	Floranthene	0.01	0.03	18	10, 6, 12
Py	Pyrene	0.05	0.15	7	11, 6, 15
B(a)A	Benzo(a)athracene	0.05	0.15	9	6, 8, 13
Chy	Chrysene	0.05	0.15	10	12, 7, 16
B(b)F	Benzo(b)fluoranthene	0.05	0.15	12	15, 5, 20
B(k)F	Benzo(k)fluoranthene	0.05	0.15	19	11, 8, 18
B(a)P	Benzo(a)pyrene	0.05	0.15	11	11, 5, 16
IP	Indeno(1,2,3-cd)pyrene	0.05	0.15	20	13, 8, 18
BDA	Dibenz(a,h)anthracene	0.10	0.30	15	10, 6, 15
B(ghi)P	Benzo(g,h,i)perylence	0.10	0.30	8	9, 8, 14

Reproducibility relative standard deviation (RSD_R_; *n* = 12) of 1 µg/kg, 5 µg/kg, and 10 µg/kg standard value.

## Supplementary Materials

The following are available online at http://www.mdpi.com/2304-8158/7/12/201/s1, Figure S1: The chromatogram of 18 PAH compounds, Table S1: Comparison of recoveries (%) between variable solvent evaporation methods.
