# Supplementary material for: Simultaneous Determination of 18 Polycyclic Aromatic Hydrocarbons in Daily Foods (Hanoi Metropolitan Area) by Gas Chromatography–Tandem Mass Spectrometry
Source: Foods. 2018 Dec 8;7(12):201. doi: 10.3390/foods7120201 (PMC6306911; doi:10.3390/foods7120201)
Supplement: Supplementary file 1 [file foods-07-00201-s001.pdf]

Supporting information

Simultaneous Determination of 18 Polycyclic Aromatic Hydrocarbons in Daily Foods (Hanoi Metropolitan Area) by Gas Chromatography–Tandem Mass Spectrometry

Thanh-Thien Tran-Lam[1], Yen Hai Dao[1], Lien Kim Thi Nguyen[1], Hoi Kim Ma[2], Hai Nguyen Tran[3*], and Giang Truong Le[1*]

[1]Institute of Chemistry, Vietnam Academy of Science and Technology (VAST), Hanoi, Vietnam
[2]University of Science, Vietnam National University HCMC, Ho Chi Minh city, Vietnam
[3]Institute of Research and Development, Duy Tan University, Da Nang, 550000, Vietnam

*Correspondences: hoasinhmoitruong.vast@gmail.com (Giang Truong Le) and trannguyenhai@duytan.edu.vn (Hai Nguyen Tran)

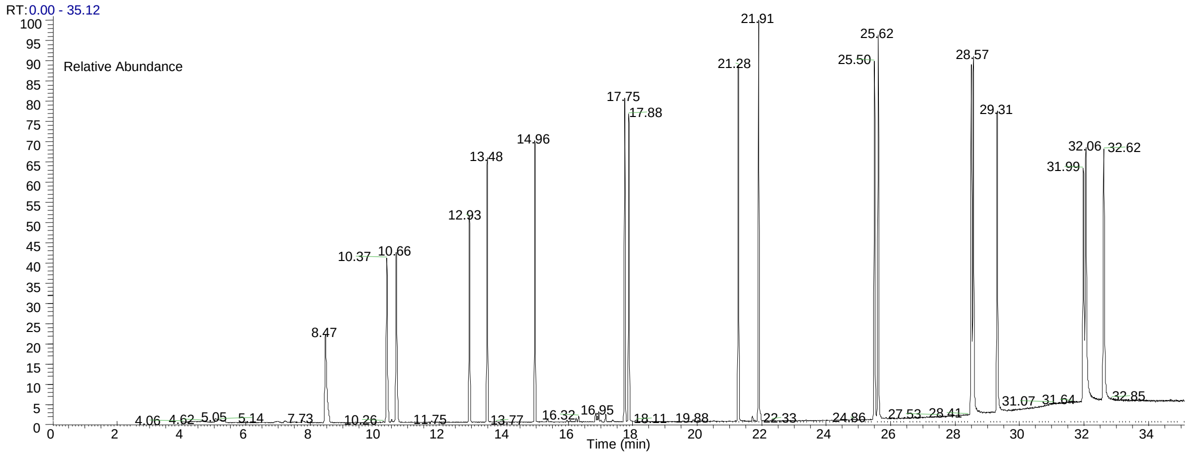

Figure S1. The chromatogram of 18 PAH compounds

Table S1. Comparison of recoveries (%) between variable solvent evaporation methods

Abbr.M	Compounds	Three methods		
		**Ambient**	**Cold**	**Rotovap**
NaP	Naphthalene	10.0±1.7	71.1±5.2	5.0±4.1
M2N	2-Methylnaphthalene	33.2±1.7	83.9±2.6	14.3±7.9
M1N	1-Methylnapththalene	34.0±2.7	84.5±3.1	9.2±5.8
ACNP	Acenaphthylene	78.9±2.8	101.2±4.2	12.2±8.7
ACP	Acenaphthene	81.4±3.6	106.2 ±4.5	23.4±9.4
FL	Fluorene	89.8±3.8	106.1±4.2	44.3±11.3
PHN	Phenalthrene	95.3±3.3	94.1±1.2	69.4±7.7
AN	Anthracene	98.4±1.2	99.1±5.3	52.8±4.1
FLA	Floranthene	99.6±5.3	100.9±2.3	61.1±3.9
Py	Pyrene	105.3±2.3	104.7±2.2	59.6±3.4
B(a)A	Benzo(a)athracene	108.3±2.2	105.8±3.3	59.4±4.4
Chy	Chrysene	102.4±3.3	106.0±3.4	55.9±3.5
B(b)F	Benzo(b)fluoranthene	106.9±3.4	108.7±5.5	54.9±5.6
B(k)F	Benzo(k)fluoranthene	103.5±5.5	101.5±5.5	54.8±6.8
B(a)P	Benzo(a)pyrene	108.1±5.5	105.6±0.5	55.4±5.8
IP	Indeno(1,2,3-cd)pyrene	107.8±4.7	101.0±3.6	48.3±3.9
BDA	Dibenz(a,h)anthracene	108.3±7.1	96.9±1.6	60.9±4.9
B(ghi)P	Benzo(g,h,i)perylence	104.8±6.7	110.6±0.9	47.2±9.9

Note: Average value ± standard deviation (SD); *n* = 5.
